# Do Routine Postoperative Radiographs Influence the Management of Distal Radius Fractures Following Volar Locking Plate Fixation?

**DOI:** 10.7759/cureus.73960

**Published:** 2024-11-18

**Authors:** Mohammed Tayyem, Nizar Ismail, Khaled F Al-Kharouf, Humam Jundi, Pallavi Singh, William Leech, Thomas Evans, Mahmoud Elmesalmi, Ramesh Chennagiri

**Affiliations:** 1 Trauma and Orthopaedics, Buckinghamshire Healthcare NHS Trust, Aylesbury, GBR; 2 Trauma and Orthopaedics, St George's University Hospitals, London, GBR

**Keywords:** distal radius fractures, post-operative imaging, postoperative radiographs, revision rate, volar locking plate, wrist fracture

## Abstract

Background: Distal radius fractures (DRFs) are a common orthopaedic injury, often requiring surgical intervention. Routine postoperative radiographs are frequently obtained after surgical fixation to ensure adequacy of fixation and rule out early complications, yet their necessity remains unclear. Through this study, we tried to evaluate the impact of routine postoperative radiographs on the management of DRFs. The objective was to determine whether routine postoperative radiographs are necessary for the effective management of patients following surgical fixation of DRFs using volar locking plates.

Methods: A review of 176 patients who underwent distal radius open reduction and internal fixation with volar locking plates at a UK district general hospital was conducted over a period of two years. Data on patient demographics, fracture characteristics, postoperative imaging new findings, and management changes were collected and analysed. The primary outcome measure was the rate of reoperation based on new findings in the routine postoperative radiographs.

Results: Routine postoperative radiographs were obtained in all the cases, with only 1% (one patient) requiring reoperation based on the presence of new findings on the postoperative radiographs. Approximately 8% (12 patients) experienced a change in their management in the form of prolonged cast immobilization.

Conclusion: Routine postoperative radiographs for DRFs with open reduction internal fixation may have limited impact on management decisions. The study highlights the potential overutilization of postoperative radiographs, leading to increased healthcare costs and radiation exposure. Based on the study's findings, a case-by-case approach, considering fracture type, associated injuries, and clinical indications, is advocated. Reducing the use of routine radiographs could save resources and reduce unnecessary radiation exposure without compromising patient care.

## Introduction

With an incidence of around 220 per 100,000 people, distal radius fractures (DRFs) are the most common fracture in adults [1,2]. They are estimated to be responsible for around 2.5% of visits to emergency departments annually [3]. DRFs are common and costly to healthcare systems like the NHS if poorly managed [4]. Thousands of operative DRF fixations are carried out annually in the UK [5,6]. Most of these patients receive routine radiographs in the first two weeks after surgery to assess the effectiveness of surgical treatment and rule out missed injuries [7].

A survey revealed that up to 80% of hand surgeons ordered radiographs during the first postoperative follow-up without symptoms guiding them [8]. The average cost of a set of wrist radiographs in the UK is around £40 [9]. Often, multiple radiographs are obtained [8], and this cost can be significantly higher in countries like the United States, where a wrist radiograph could cost approximately $85 [10].

Generally, patients requiring radiographs also need a clinic appointment, adding further financial burden due to the added time per appointment. However, it is difficult to quantify how significant this burden is, as no comprehensive data is available. No clear or accepted guidelines suggest whether these radiographs are necessary for postoperative DRF care [8]. There have been a few studies recently to study the utility of routine postoperative radiographs after fixation of common fractures. Our study, conducted in a UK district general hospital (DGH), aims to establish whether routine postoperative radiographs are helpful in managing patients with distal radius fractures and whether they lead to a useful change in treatment plans.

## Materials and methods

A retrospective review of patients treated for distal radius fractures at Stoke Mandeville Hospital (Buckinghamshire Healthcare NHS Trust) was completed. Patients were identified using the electronic patient record and theatre scheduling system (Bluespier; Clanwilliam, Ireland). The reviewed patients were treated with volar locking plates between January 1, 2018, and December 31, 2019. The included patients were all over 16 years old, had DRFs treated with plates and screws, and had intraoperative images saved on PACS (picture archiving and communication system, the electronic database for imaging). Patients with concurrent fractures (e.g., scaphoid fractures), patients younger than 16, and patients treated with other modalities (K-wires, external fixators) were excluded. The data were extracted from Evolve, the electronic database, and PACS at Stoke Mandeville Hospital.

The following information was collected: patient age and gender, type of injury (open, closed, intra- or extra-articular), total number of postoperative images, total number of postoperative follow-up appointments, and the surgeon's grade. This information was collated in Microsoft Excel (Microsoft Corporation, Redmond, WA), and descriptive analysis with cross-tabulation was performed. A total of 181 patients were initially included in the study; five patients were excluded as they did not meet the inclusion criteria.

Postoperative radiographs at the first clinic visit within the first two weeks were reviewed by two authors (MT, NI) to identify positive findings, which were defined as loss of reduction, unsatisfactory reduction, intra-articular screws, prominent plates, or screws backing out. After reviewing radiographs, management changes were documented. Management changes were defined as any deviation from the postoperative management plan, such as requests for CT scans, prolonged immobilization, or reoperations. The primary outcome measure was the reoperation rate after positive findings on postoperative imaging. Secondary outcome measures included changes in the management plan.

The study adhered to the Caldicott principles and was submitted to the audit department at Stoke Mandeville Hospital for ethical consideration, and was subsequently presented in the monthly departmental audit meeting.

## Results

A total of 176 patients (142 female patients, 34 male patients) were included in the study, all of whom underwent distal radius open reduction and internal fixation with plates and screws. Patients' age ranged from 17 to 87 years, with a mean age of 58.5 years. Of the fractures, 139 were intra-articular, and 37 were extra-articular. Most injuries were closed (169 closed, 7 open fractures), and five patients had associated distal ulna metaphyseal fractures. These five were treated either operatively or conservatively.

The grade of the first surgeon in each operation was noted, with 71 procedures supervised by a hand consultant, 49 by general trauma and orthopaedics consultants, and 50 by registrars. Twenty-three of the 176 patients were found to have new positive findings in their postoperative follow-up radiographs (Table 1). They were categorized as the positive findings group (loss of reduction, unsatisfactory reduction, intra-articular screws, prominent plates, or screws backing out). The rest of the study population showed stable postoperative radiographs compared to intraoperative images.

**Table 1:** New positive findings on postoperative distal radius radiographs (performed in the first two weeks after surgery)

Positive finding	Number of patients
Unsatisfactory or loss of reduction	18
Screw backing out	2
Intra-articular screw/pegs	3
Prominent plate	2

Twelve out of 23 patients experienced a change in their treatment plan after a positive finding on postoperative radiographs (Table 2). Only one patient required acute reoperation due to a displaced ulna fracture, resulting in re-fixation of the ulna (<1%). Three patients required CT scans, seven patients experienced a prolonged period of immobilization (more than two weeks), and two patients required the removal of metalwork after fracture union due to plate prominence and flexor tendon irritation after more than six months of the original procedure.

**Table 2:** Change in the treatment plan in the first postoperative outpatient clinic appointment after reviewing radiographs in patients with new radiographic findings

Change in treatment of plan	Number of patients
Acute reoperation	1
Removal of metalwork after fracture union	2
Prolonged immobilization	7
CT scan	3

A total of 363 outpatient follow-up appointments and 285 radiographs were arranged for the study population, of which 67 follow-ups and 57 radiographs were for patients in the positive findings group (Table 3).

**Table 3:** Number of follow-ups and postoperative radiographs in each group

	Negative postoperative X-ray findings group	Positive postoperative X-ray findings group
Number of patients	153	23
Total number of follow-ups	296	67
Total number of radiographs	218	57
Average number of follow-ups	1.93	2.91
Average number of radiographs per patient	1.42	2.48

## Discussion

Routine radiographs after distal radius surgical fixation with plates and screws are common practice. Bohl et al. estimated that approximately 2.6 ± 1.0 radiographs are obtained per asymptomatic patient [8], while Huffaker et al., in a retrospective review, observed an average of 2.9 radiographs per patient [11]. Our retrospective study demonstrated that 99% of patients had postoperative radiographs, with an average of 1.63 radiographs per patient postoperatively.

Few studies have evaluated whether routine postoperative radiographs are necessary or lead to a change in the management plan. Sharma et al. found that radiographs resulted in a 2.4% change in management, with a less than 1% risk of reoperation [12]. Similarly, Ghattas et al. reported that most postoperative radiographs did not change the management plan [13]. Other studies reported comparable results [9].

Our results showed that the reoperation rate was less than 1%; this a highly significant finding indicating that the yield of such practice was limited. In our study, this was attributed to the displacement of the unfixed associated distal ulna shaft fracture, rather than a complication involving the radius. Other changes in the management plan were evident in about 8% of cases in our series. Most of these changes in management, such as extended immobilization, were likely unnecessary. Research indicates that prolonged immobilization for distal radius fractures treated with plates and screws can be rather harmful, with no evidence of better outcomes [14]. Watson et al. suggested that extended immobilization may result in adverse effects instead of benefits [15].

The indications for CT scans in three cases were to rule out intra-articular screws. However, in all cases, smooth pegs were used, which are less concerning for intra-articular penetration compared to screws [16]. Ultimately, none of the three patients required further intervention as they were asymptomatic during the recovery period.

Patients with new postoperative changes on postoperative radiographs findings often required more follow-up visits and subsequent radiographs, adding to healthcare costs and exposing patients to more radiation. Many of these additional follow-ups were likely unnecessary, prompted by concerning findings on the initial postoperative radiographs.

To justify routine postoperative radiographs, the benefits of radiographs must outweigh resource costs and radiation risk to patients [17]. However, our findings showed that only one patient with concurrent ulna fractures had a significant need for early radiograph imaging. Due to the limited number of patients who had revisions, we were unable to identify a scoring system to determine which patient needed routine radiographs. Other studies, including that by Sharma et al., demonstrated that intra-articular fractures are more likely to result in management changes based on radiographs [12].

Due to the limited value of routine postoperative radiographs, clinical decision-making should be individualized, considering factors such as the fracture type, any related injuries, and the potential implications of delaying a reoperation. We believe that postoperative radiographs should only be performed when there is a clear clinical indication, based on the original fracture pattern, stability of fixation, and the presence of new symptoms.

This study also has limitations. First, the retrospective nature of the study gives a slightly skewed and limited data set; a prospective study would give more accurate results moving forward. Second, the study population being from a single centre, essentially from one DGH, gives a limited insight into the role of postoperative follow-up in distal radius fracture management and certainly cannot be used to accurately reflect management practice nationally. The relatively small patient cohort also limits the project’s significance. Finally, the secondary outcome measure, i.e., deviation from the original management plan, is largely dependent on the subjective opinion of the clinician reviewing the patient and this may limit the clinical significance of this measure.

Moving forward, development of this project to become a multi-centre study with a much larger patient cohort would yield stronger, more statistically robust results. Furthermore, expanding the project to include prospective, multi-centre studies would help offer a much more significant insight into the shape of current practice in the UK and could help shape policy and guidelines and influence practice in the sector. It could also potentially identify areas where significant savings in terms of resources and improved efficiency can be successfully implemented.

## Conclusions

We can conclude that the value of routine early imaging in the postoperative period after distal radius plating is limited. We suggest that routine radiographs should not be a part of the postoperative protocol, and that the plan to request radiographs during the first postoperative clinic visit should be based on symptoms, fracture pattern, and stability of fixation. National policies should reflect this approach, advocating for more selective imaging based on clinical indicators rather than routine practice.
